# Editorial: Cardiogenic shock: basic and clinical consideration, volume II

**DOI:** 10.3389/fcvm.2024.1524631

**Published:** 2024-11-22

**Authors:** Umberto Di Dedda, Indranee Rajapreyar, J. Eduardo Rame, Clement Delmas, Paolo Meani

**Affiliations:** ^1^ Cardiac Anaesthesia and Intensive Care, IRCCS Policlinico San Donato, Milan, Italy; ^2^ Division of Cardiovascular Disease, Department of Medicine, University of Alabama at Birmingham, Birmingham, AL, United States; ^3^ Department of Medicine, Division of Cardiology, Thomas Jefferson University Hospital, Philadelphia, PA, United States; ^4^ Intensive Cardiac Care Unit, Department of Cardiology, Rangueil University Hospital, Toulouse, France; ^5^ Department of Cardiothoracic Surgery, Heart and Vascular Centre, Maastricht University Medical Centre, Maastricht, Netherlands; ^6^ Faculty of Health, Medicine and Life Sciences, Maastricht University, Maastricht, Netherlands

**Keywords:** cardiogenic shock, organ cross talk, cardiogenic shock phenotypes, mechanical circularity support, acute cardiovascular care

**Editorial on the Research Topic**
Cardiogenic shock: basic and clinical consideration, volume II

Cardiogenic shock (CS) is a life-threatening condition leading to poor prognosis and high morbidity. The related inadequate cardiac output carries to tissue hypoperfusion, hypoxia and multi-organ failure. The CS etiology has been profoundly changing. Thanks to the widespread availability of percutaneous coronary intervention, the acute myocardial infarction is nowadays better treated. Therefore, the contribution of myocardial infarction, which was highly predominant, has decreased with an increasing proportion of other etiologies (1).

Furthermore, the availability of advance therapies, including mechanical support offer valuable options for infarct related complications, such as muscle wall rapture. The emerging evidence underpinning the mechanical circulatory support use in ventricular septal and free wall rupture as a bridge to surgery repair has been overcoming the previous therapeutical limitations (2). Additionally, the ability to predict and prevent profound CS by early support implementation, either pharmacological or mechanical, currently represents a concrete approach in such conditions considered hopeless in the past (Wang et al.). As a result, even if the clinical impact related to acute myocardial infarction has been dramatically changing, the available weapons are clearly posing new challenges also in the ischemic CS patients.

Besides the different etiology behind CS, the growing patients' complexity and heterogenicity highlights the role of potential risk modifiers (3), as well as the intimate cross-talks with other organs, such as brain and liver (4).

Risk modifiers such as age, the presence of systemic inflammatory response syndrome, acute kidney injury, and other noncardiac organ failure, severe acidosis, echocardiographic findings, lactates and invasive hemodynamics derived from pulmonary artery catheter have been extensively investigated (3). In addition, the presence of arrhythmic triggers, either supra-ventricular or ventricular, may also strongly affect patients' outcomes. On one hand, the relative shortage of anti-arrhythmic drugs without significant side effects in terms of ventricular function may limit the treatment effectiveness. On the other hand, arrhythmias are important markers of disease severity, selecting the most demanding scenarios. Moreover, based on the aforementioned therapeutical limitations, when arrhythmic patterns occur, patients often end up to heart transplantation or durable mechanical supports (such as LVAD or Bi-VAD) (Cherbi et al.).

Furthermore, the exploration of interorgan cross-talk should be also considered when facing CS patients. For instance, the profound interdependence between neurological and cardiac health, especially in inflammatory diseases, emphasizes the brain-heart relationship, where neurological stressors might influence cardiac dynamics and trigger the development of cardiac dysfunction and, therefore, precipitate CS. Myocardial damage is increasingly observed in association with acute neurologic disorders, highlighting how neurological conditions can directly impact heart function. Neurologic insults, such as stroke, epilepsy, traumatic brain injury, and multiple sclerosis, are known to trigger cardiac events due to intense autonomic nervous system activation (5, Brandner et al.). This autonomic dysregulation, often through sympathetic nervous system surges, can lead to myocardial injury, arrhythmias, or stress-induced cardiomyopathies like Takotsubo syndrome (TTS). The reverse TTS variant, although uncommon, is noteworthy in the context of neurological disorders and has been documented in limited case reports. Evidence linking MS, particularly with brainstem lesions, to TTS onset, reinforces the role of the central nervous system in modulating cardiac stress responses (Brandner et al., 6). Given the intricate mechanisms linking brain inflammation and cardiac function, further research is needed to unravel the pathophysiology underlying neurocardiac syndromes and their rare presentations, ensuring that early signs of cardiac dysfunction in neurologically compromised patients do not go unnoticed.

Yet, haematologic disorders may involve a number of organ tissues, including the heart. Hypereosinophilia may lead to a significant rare cardiac involvement with potentially life-threatening complications, such as CS. The cardiac dysfunction is often under-recognized, and carries a poor prognosis. The early identification and phenotypization of inflammatory infiltrates in endomyocardial biopsies, plays a pivotal role and facilitates a targeted therapy improving the clinical course significantly (7, Placidi et al.). Therefore, establishing standardized recommendations for interpreting uncommon findings in CS patients could reorient the clinical approach, enhance outcomes, and minimize the diagnostic/therapeutic uncertainties surrounding the heterogeneous scenario of CS.

As a consequence, the increased patients’ complexity and morbidity, as well as the broad etiology spectrum prompt an urgent need of re-assessing CS management models. As a matter of fact, the technology progress currently provides several tools which might better guide the CS treatment with increasing levels of invasiveness (8). Nevertheless, little is known about how to pragmatically define centers with different levels of care. Furthermore, defined features, such as distinct demographics, practice patterns and mechanical supports availability may ultimately impact on clinical outcomes (Villela et al.). The rising interest on “hub and spokes” system seems to be an effective strategy which needs to be urgently implemented in our CS network. Moreover, the feasibility of the current CS hospital models should be also urgently evaluated by the upcoming research.

To conclude, the growing patients' complexity combined with the aforementioned CS etiology unmasking, as well the development of advanced pharmacological and mechanical therapies have been posing new CS treatment challenges. In fact, the traditional approach to CS relies on simply assessing clinical and hemodynamic profiles. However, the biological and molecular diversity within CS calls for a more comprehensive approach that integrates host-response biomarkers, defining pathophysiological events of CS include (including) subclasses with distinct underlying biological/molecular mechanisms (9). This, exploring biomarkers of damage in cardiac and extra-cardiac perfusion-sensitive organs, targeting metabolomic pathways, and examining endothelial function and dysfunction in the dynamic microcirculation during inflammation may reveal the biological granularity underlying the causes and effects of shock states. As we shift focus from macro to microenvironments, biomarker-driven endotypes—often imperceptible to bedside clinicians—may expand our understanding of CS heterogeneity (10). Given these circumstances, further studies addressing the heterogenicity of CS sub-phenotypes are urgently needed.
